# GV1001 interacts with androgen receptor to inhibit prostate cell proliferation in benign prostatic hyperplasia by regulating expression of molecules related to epithelial-mesenchymal transition

**DOI:** 10.18632/aging.202242

**Published:** 2021-02-04

**Authors:** Yejin Kim, Dahae Lee, Hyejung Jo, Cheolhyeon Go, Jongwon Yang, Dongmin Kang, Jae Seung Kang

**Affiliations:** 1 Department of Anatomy, Seoul National University College of Medicine, Seoul, Republic of Korea; 2 Institute of Allergy and Clinical Immunology, Seoul National University Medical Research Center, Seoul, Republic of Korea; 3 Department of Psychological and Brain Sciences, College of Arts and Sciences, Boston University, Boston, MA 02215, USA

**Keywords:** GV1001, benign prostate hyperplasia, androgen receptor, dihydrotestosterone, epithelia-mesenchymal transition

## Abstract

Prostate cell proliferation, driven by testosterone, is a major characteristic of benign prostatic hyperplasia (BPH). GV1001, a human telomerase reverse transcriptase catalytic subunit, is an injectable formulation used as a cancer vaccine. It functions as a cell penetrating peptide to regulate cell proliferation. Here, we found that GV1001 effectively suppressed proliferation of prostatic stromal myofibroblasts (WPMY-1) and prostatic epithelial cells (RWPE-1 and WPE-NA22) treated with dihydrotestosterone. Also, GV1001 bound to androgen receptors (ARs) in the cytosol of stromal and epithelial cells. In an experimental animal model implanted with an infusion pump for spontaneous and continuous release of testosterone, revealed that GV1001 reduced prostatic hypertrophy and inhibited the cell proliferation and the expression of Ki67, proliferating cell nuclear antigen, and prostate specific antigen. In addition, GV1001 prevented fibrosis of the prostate by downregulating expression of prostatic epithelial-mesenchymal transition (EMT)-related proteins such as transforming growth factor (TGF)-β, Snail, Slug, N-cadherin, and Vimentin, and by up-regulating E-cadherin. Taken together, these results suggest that GV1001, which suppresses TGF-β-mediated EMT by outcompeting testosterone for binding to AR, is a potential therapeutic drug for BPH accompanied by prostatic fibrosis.

## INTRODUCTION

Benign prostatic hyperplasia (BPH) is a slow, progressive and common disease in aging men; prevalence increases from 25% among men aged 40–49 years to more than 80% in men aged 70–79 years [1]. BPH is characterized by an enlargement of the transitional prostatic zone of the prostate, which compresses the urethra and causes urinary obstruction and lower urinary tract symptoms [2, 3]. In terms of histological definition, a hallmark of BPH is non-malignant and aberrant proliferation of epithelial and stromal cells. The etiology of BPH is highly complex and the underlying mechanisms are poorly understood; however, embryonic reawakening, imbalance of hormone levels, increased transforming growth factor (TGF)-β signaling, stem cell defects, chronic inflammation, and other unidentified factors have been suggested [3].

Androgenic hormones testosterone and dihydrotestosterone (DHT) play an important role in development of BPH. Men with impaired androgen activity due to genetic disease or castration before puberty do not develop BPH [4]. Furthermore, medical therapies such as androgen withdrawal and inhibition of testosterone metabolism yield clinical responses in patients with symptomatic BPH [5, 6]. Over 90% of total prostatic androgen is in the form of DHT, which is derived from testosterone by the action of 5α-reductase [7]. Testosterone and DHT bind to androgen receptors (ARs) expressed in prostatic stromal and epithelial cells. DHT is a more potent androgen than testosterone because it has a higher affinity for AR [8]. AR binds a complex of heat shock protein (HSP) chaperones, which ensures that it retains the correct conformation for DHT binding [3]. The androgen/AR complex is translocated to the nucleus, where it binds to specific DNA-binding sequences such as androgen response elements, resulting androgen-dependent gene expression [4]. Androgen/AR signaling affects initiation and progression of BPH by altering expression of various growth factors in a paracrine and autocrine manner; these factors promote growth of prostatic epithelial and stromal cells, accompanied by increasing expression of epithelial-mesenchymal transition (EMT)-related molecules such as TGF-β, ZEB-1, and Snail [9, 10]. In addition, it regulates development of BPH by regulating the inflammatory environment and immune cell infiltration. Therefore, targeting androgen/AR signaling could be a major therapeutic approach for BPH [11].

GV1001 is a peptide corresponding to amino acids 611–626 of reverse transcriptase subunit of telomerase (hTERT). Initially, it was developed as a peptide vaccine against various cancers, and was proven safe in several clinical trials of patients with pancreatic cancer, non-small cell lung cancer, melanoma, hepatocellular carcinoma, and prostate cancer [12–17]. Recently, we reported that a combination of GV1001 and gemcitabine resulted in significant loss of fibrosis in tumor tissue, as well as increasing tumor cell death [18]. Kim et al., reported that GV1001 reduced levels of 5α-reductase in prostate tissue from experimental animals with BPH caused by testosterone injection after castration [19]. In addition, the drug was tested in phase II clinical trials of patients with BPH [20]. However, the mechanism by which GV1001 decreases and regulates the symptoms of BPH at a cellular level (i.e., regulating prostatic epithelial and stromal cell proliferation) is unclear. Most animal models of BPH are based on subcutaneous (s.c.) injection of testosterone after castration, not on spontaneous release of testosterone *in vivo*. Here, we developed a testosterone-induced BPH mouse model using a micro-osmotic pump that facilitates spontaneous release of testosterone *in vivo.* We then investigated the mechanisms and therapeutic effects of GV1001 against BPH.

## RESULTS

### GV1001 suppresses DHT-induced proliferation of prostate epithelial and stromal cells

**Figure 1:**
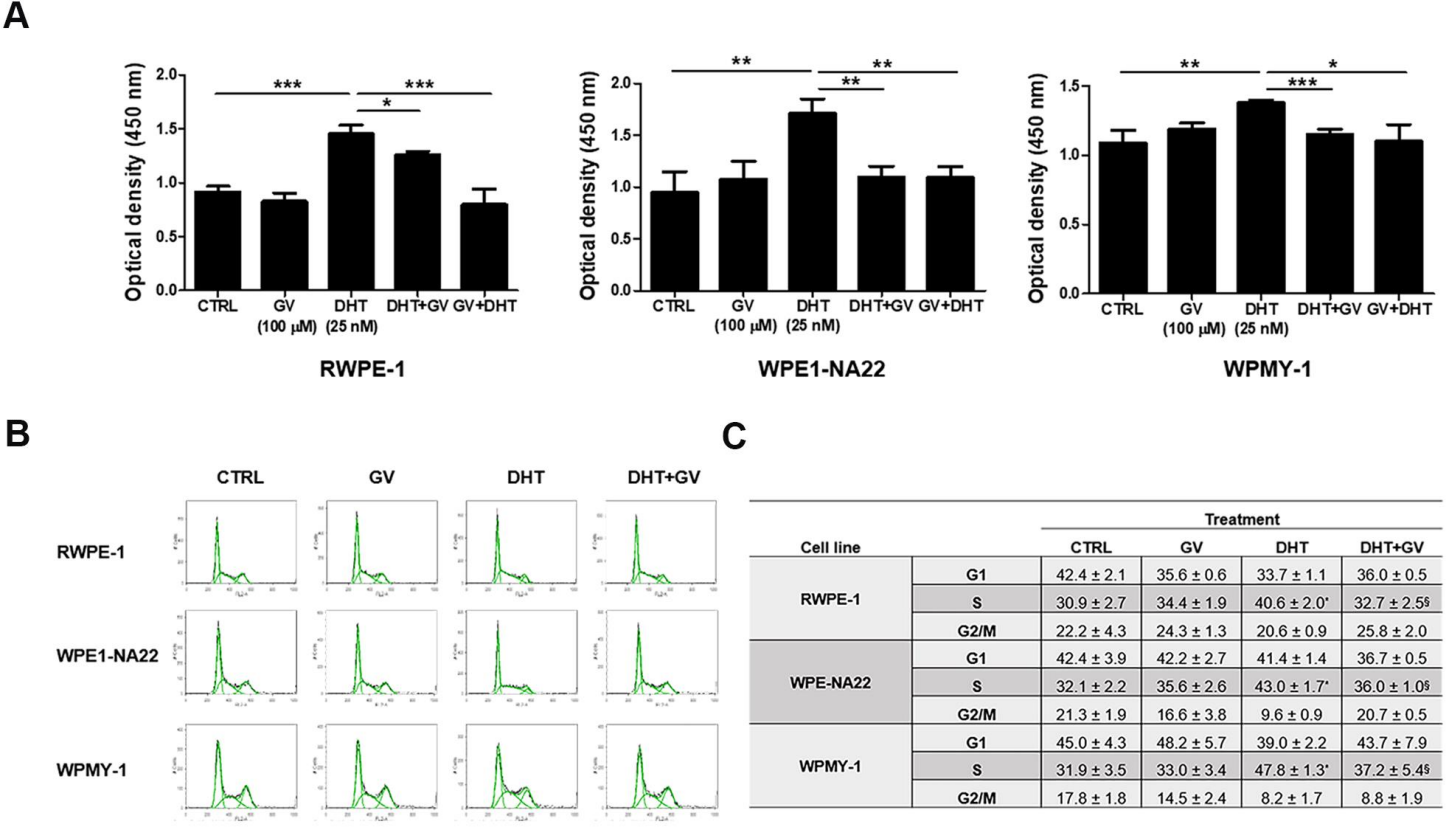
**Anti-proliferative effects of GV1001 on prostatic epithelial and stromal cells.** (**A**) Prostatic epithelial cell lines, RWPE-1, WPE1-NA22, and stromal cell line WPMY-1 were treated with dihydrotestosterone (DHT, 25 nM) or GV1001 (GV, 100 μM). Cells were exposed to GV1001 1 h before DHT treatment (GV + DHT) or co-treated with both DHT and GV1001 (DHT + GV). After 48 h, proliferation was accessed in a CCK-8 assay (n=5). ***p<0.001, **p<0.01, *p<0.05. Data are expressed as the mean ± SD. (**B**, **C**) RWPE-1, WPE1-NA22, and WPMY-1 cells were co-treated with DHT (25 nM) and GV1001 (100 μM) for 48 h and cell cycle analyzed by flow cytometry after PI staining. Data are presented in a histogram (**B**) and a table (**C**). ^*^ = Significantly different from the control (p<0.01). ^§^ = Significantly different from the DHT group (p<0.05).

To investigate the down-regulatory effects of GV1001 on prostatic hyperplasia, we exposed DHT to prostatic epithelial cell lines, RWPE-1, WPE1-NA22 and prostatic stromal myofibroblast cell line, WPMY-1 in the presence/absence of GV1001. A CCK-8 assay revealed that DHT-induced proliferation of RWPE-1 and WPMY-1 in a dose-dependent manner (Supplementary Figure 1A, 1B). Also, pre- or co-treatment with GV1001 effectively suppressed the increased proliferation of RWPE-1, WPE1-NA22, and WPMY-1 cells (Figure 1A). To clarify the underlying mechanism, we examined changes in the cell cycle distribution of cells exposed to GV1001. We found that DHT increased the percentage of prostatic epithelial and stromal cells at S phase, and that this was suppressed by GV1001 (Figure 1B, 1C). Taken together, these results show that GV1001 suppressed DHT-induced proliferation of prostatic epithelial cells and stromal cells by downregulating the cell cycle at S phase.

### GV1001 binds to AR expressed by prostate epithelial and stromal cells

**Figure 2:**
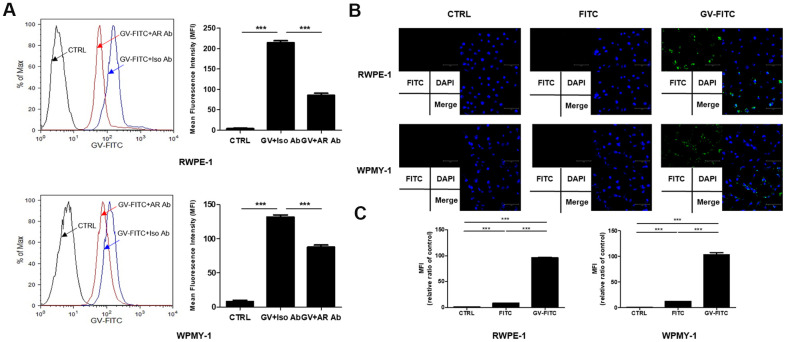
**Interaction of GV1001 with AR in prostatic epithelial and stromal cells.** (**A**) RWPE-1 and WPMY-1 cells were pre-incubated for 1 h with an anti-AR antibody or an isotype control antibody, followed by FITC-conjugated GV1001 (GV-FITC) for 30 min. Then, GV1001-bound cells were analyzed by flow cytometry. The mean fluorescence intensity (MFI) of FITC in RWPE-1 and WPMY-1 cells is presented in the graph. ***p<0.001. (**B**) GV-FITC was applied to RWPE-1 and WPMY-1 cells, and internalization of the peptide was analyzed by fluorescence microscopy. Scale bar: 125 μm. (**C**) Interaction of GV1001 with AR was confirmed by co-immunoprecipitation.

Since AR antagonists are used widely to treat BPH, we next examined whether GV1001 binds to AR expressed by prostatic epithelial and stromal cells. We found that binding of FITC-conjugated GV1001 to AR, expressed by RWPE-1 and WPMY-1 pre-treated with an anti-AR antibody, was lower than that to cells treated with an isotype control (Figure 2A). Thus, binding of GV1001 to AR in the cytoplasm of prostate cells was inhibited by the anti-AR antibody, suggesting that GV1001 combines with AR. GV1001 penetrates the membrane of cells, including both primary and tumor cells [21]; therefore, we subjected cells to internalization assays followed by fluorescence microscopy. GV-FITC showed higher cell penetrating activity that the control (Figure 2B). Next, to confirm the interaction between GV1001 and AR under physiological conditions, we attempted to identify GV1001-interacting proteins by co-precipitation with an anti-AR antibody. The interactions with GV1001 were confirmed in a microplate reader. The mean fluorescence intensity (MFI) of GV-FITC was much higher than that of the negative or FITC-only controls (Figure 2C). Taken together, these data support an interaction between GV1001 and AR.

### GV1001 relieves prostate hyperplasia in a murine model of testosterone-induced BPH

**Figure 3:**
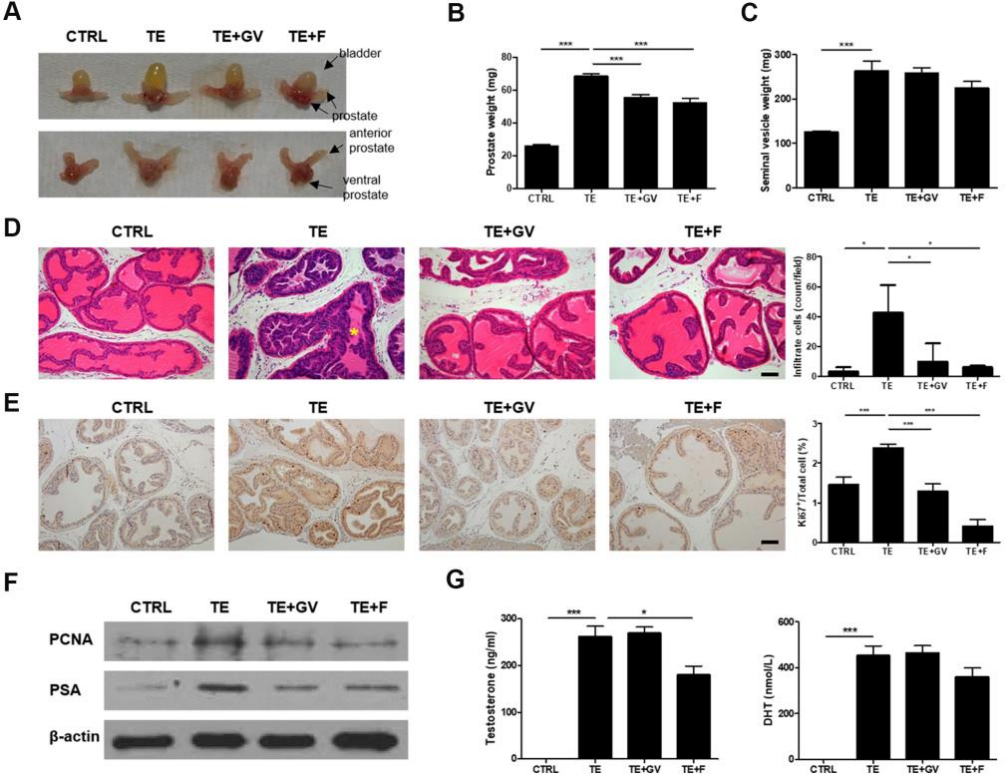
**Decreased prostate cell proliferation and immune cell infiltration by GV1001 in BPH mice.** BPH mice received either GV1001 (GV, 250 μg/head) or finasteride (F, 100 mg/kg) for 2 weeks. (**A**) A representative prostate from each group is shown (n=7 mice/group). Upper panels show the urinary bladder and lower panels do not. (**B**) The weight of prostate and (**C**) seminal vesicles was measured at the end of the experiments. ***p<0.001. (**D, E**) Prostates were excised, fixed and stained (**D**) with hematoxylin and eosin (H&E) and (**E**) with an anti-Ki67 antibody. Scale bar, 100 μm. The bar graph shows the number of infiltrating immune cells and Ki67-positive cells. ***p<0.001, *p<0.05. The asterisk (*) indicates infiltrating immune cells. (**F**) Expression of PCNA and PSA in prostate cell lysates was examined by immunoblotting; β-actin was used as an internal control. (**G**) Plasma concentration of testosterone and DHT was measured using EIA kits. ***p<0.001, *p<0.05.

To verify the therapeutic effect of GV1001 *in vivo*, a mouse model for BPH was established in which testosterone enanthate (TE) was infused by a micro-osmotic pump. Enlargement of the prostate after injection of TE was suppressed by GV1001. The effect was similar to that of treatment with finasteride, a 5-alpha reductase inhibitor, which was used as a positive control (Figure 3A, 3B). There was no marked difference among TE-induced groups with respect to the weight of seminal vesicles (Figure 3C). The body weight of mice with BPH fell slightly and only recovered after treatment with finasteride (Supplementary Figure 2). Moreover, we noted hyper-proliferation of cells in the prostate, irregular shaped prostatic glands, and infiltration by inflammatory immune cells in lumen after implantation of the TE-containing micro-osmotic pump; however, these effects were ameliorated by GV1001 (Figure 3D). The results from the GV1001-treated group were similar to those of the group treated with finasteride. Greater nuclear localization of Ki67, a proliferation marker, was observed in the TE-induced BPH prostate, but this decreased in the GV1001- or finasteride-treated prostate (Figure 3E). Cell proliferation was assessed by immunoblot analysis of prostate lysates with an antibody specific for cell nuclear antigen (PCNA). In addition, prostate specific antigen (PSA) levels were measured to detect irritation or infection of the prostate in BPH [22]. Expression of PCNA and PSA in prostate tissue from mice implanted with a micro-osmotic pump containing TE was high; however, expression of both was suppressed completely by treatment with GV1001 or finasteride (Figure 3F and Supplementary Figure 5). The plasma concentration of testosterone and DHT was measured in an enzyme immunoassay (EIA) to investigate whether GV1001 affects endogenous production of testosterone and its conversion to DHT. In contrast to the effect of finasteride, GV1001 did not suppress testosterone production or its conversion to DHT in mice fitted with the osmotic pump (Figure 3G).

### GV1001 reduces TGF-β production, development of EMT, and fibrosis

**Figure 4:**
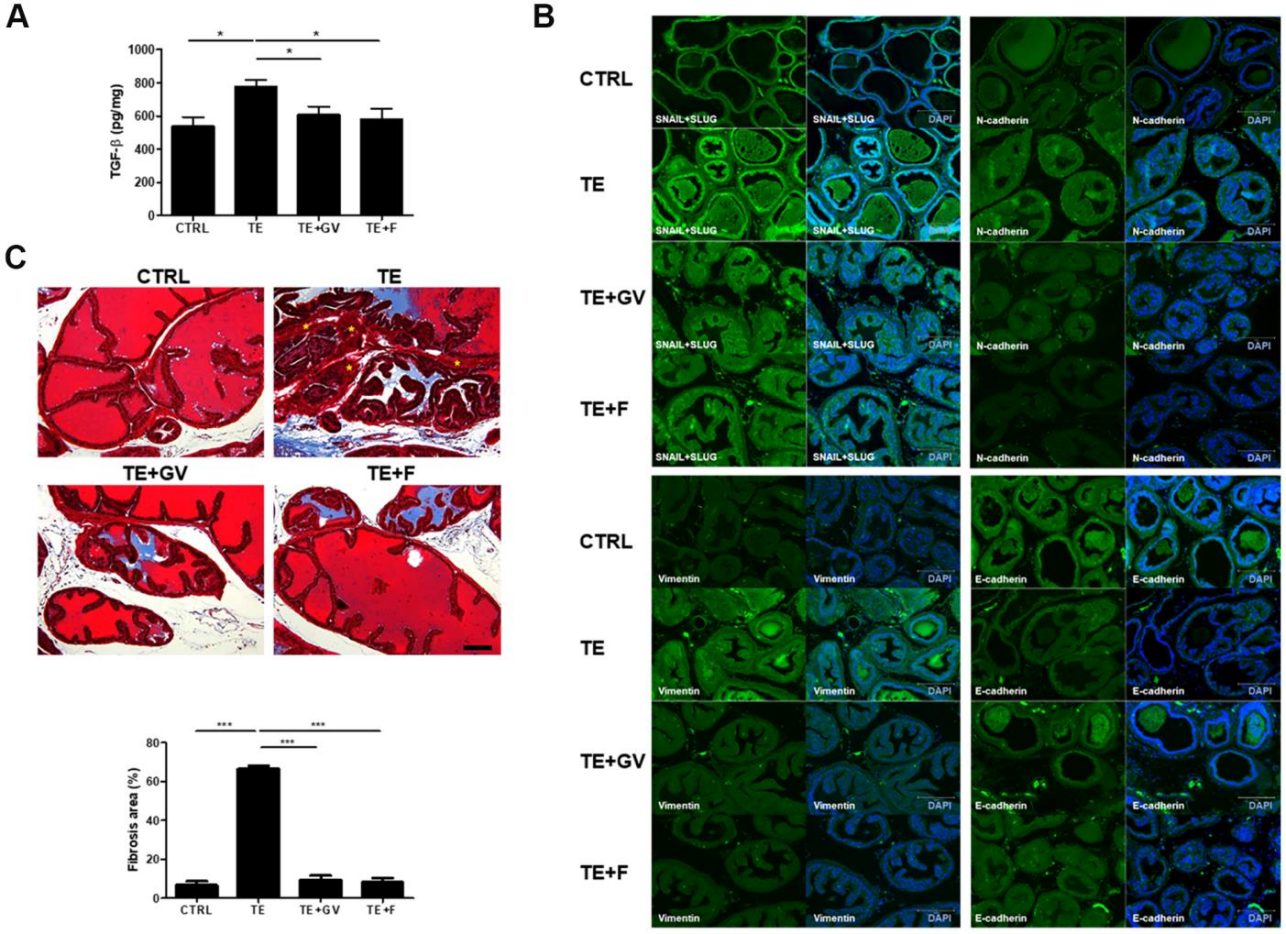
**Regulation of TGF-β production and EMT-related molecules, and alleviation of fibrosis, by GV1001 in the prostate of BPH mice.** BPH mice were treated with GV1001 (GV) or finasteride (F) for 2 weeks. (**A**) After prostates were homogenized, the level of TGF-β in the lysates was measured in an ELISA. *p<0.05. (**B**) Expression of EMT-related proteins (Snail, Slug, N-cadherin, Vimentin, and E-cadherin) in the prostate was examined by immunofluorescence. Scale bar, 150 μm. (**C**) Prostates were fixed and sections were stained with Masson’s Trichrome. Scale bar, 100 μm. Asterisks (*) indicate smooth muscle (red). Collagen fibers were stained blue. The bar graph shows the area of fibrosis stained positive with Masson Trichrome. ***p<0.001.

Androgen/AR signals affect initiation and progression of BPH by altering expression of factors involved in EMT [9, 23]. In general, TGF-β, generated by the interaction between androgens and AR, plays an important role in EMT that accompanies fibrosis in the prostate [24]. TGF-β production in mice implanted with a micro-osmotic pump containing TE increased markedly, but was suppressed effectively by GV1001 and finasteride (Figure 4A). In the case of many carcinomas, TGF-β appears to be responsible for induction or functional activation of a series of EMT-inducing transcription factors in cancer cells, notably Snail and Slug. Also, Snail represses expression of epithelial makers (*e.g*., E-cadherin) and induces expression of EMT-associated mesenchymal markers (*e.g*., N-cadherin and Vimentin) [25, 26]. To clarify the specific mechanisms by which GV1001 prevents TGF-β-induced EMT in the prostate, we examined changes in expression of Snail, Slug, N-cadherin, Vimentin, and E-cadherin. Increased expression of Snail, Slug, N-cadherin, and Vimentin in TE-induced mice was suppressed by GV1001. By contrast, expression of E-cadherin, which is related to MET, a process opposite to that of EMT, was downregulated by TE but recovered in the presence of GV1001 (Figure 4B). EMT leads to pathologic fibrosis; therefore, we next performed Masson’s Trichrome staining to examine whether GV1001 prevents TGF-β-induced fibrosis in the prostate. We found that extensive fibrosis induced by TE was prevented by GV1001 and finasteride (Figure 4C).

### GV1001 decreases TNF-α production by human peripheral blood mononuclear cells (PBMCs) stimulated with DHT

**Figure 5:**
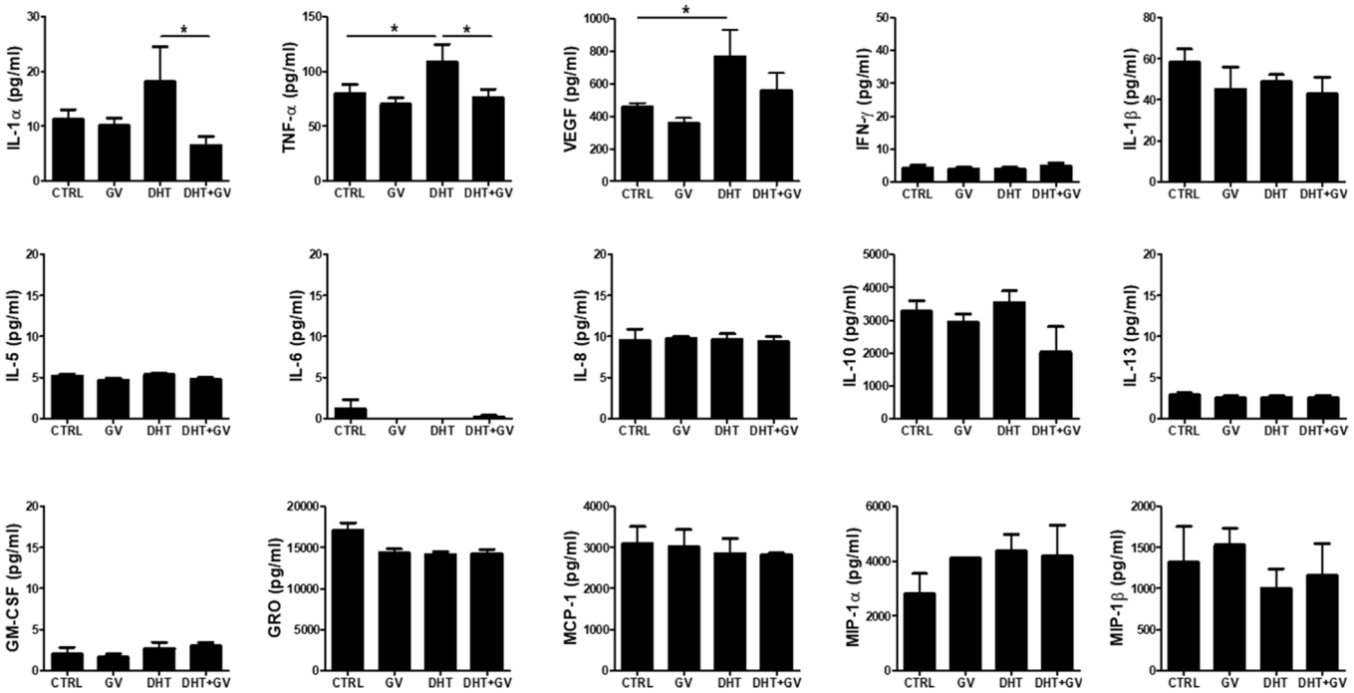
**Decreased TNF-α production by GV1001 in human peripheral blood mononuclear cells (PBMCs) stimulated with DHT.** PBMCs (2.5 × 10^6^ cells/ml) were treated with either DHT (25 nM) or GV1001 (GV, 100 μM) for 24 h and the media were collected for cytokine array analysis. The concentration of 15 cytokines (IL-1α, IL-1β, IL-5, IL-6, IL-8, IL-10, IL-13, TNF-α, GM-CSF, GRO, IFN-γ, MCP-1, MIP-1α, MIP-1β, VEGF) secreted by DHT-stimulated PBMCs was measured using a human cytokine array kit. Data are presented in the bar graph. *p<0.05.

In general, inflammatory responses are accompanied by development and progression of BPH. In fact, when human monocytic cell line THP-1 is treated with DHT, production of TNF-α increases in accordance with cell activation [27]. Therefore, we investigated the ability of GV1001 to regulate production of inflammatory cytokines in PBMCs stimulated with DHT. Overall, we examined the levels of 15 cytokines in response to DHT stimulation in the presence/absence of GV1001. We found that DHT increased the levels of IL-1α, TNF-α, and VEGF; however, GV1001 only downregulated IL-1α and TNF-α to a marked extent (Figure 5)

## DISCUSSION

GV1001, a hTERT-derived peptide, was generated as a vaccine against several cancers; it acts by inducing cancer-specific CD4/CD8 T cell immune responses that eradicate tumors and instill long-term memory [28]. Here, we report a novel function of GV1001: it effectively suppresses proliferation of prostatic epithelial cell and stromal cells *in vitro* and reduces enlargement of the prostate in an experimental mouse model of BPH. In contrast to most *in vivo* experiments regarding BPH, which were performed in animal models subcutaneously injected with testosterone after castration [29, 30], we examined the therapeutic effects and related mechanisms of GV1001 in a testosterone-induced BPH model in which mice were implanted with a micro-osmotic pump to allow spontaneous release of testosterone *in vivo* (Supplementary Figure 3). There was no effect on BPH in mice treated with GV1001 or finasteride alone (Supplementary Figure 4). This results in a situation very similar to spontaneous development of BPH in humans, meaning that our findings are applicable to humans and that our experimental models could be useful for studies on BPH drug development.

We found that GV1001 had multiple effects on prostate cells with respect to proliferation and EMT. Because EMT is crucial for the pathogenesis and progression of BPH, the mitigating effects of GV1001 on multiple factors related with EMT suggest that the drug has therapeutic potential for BPH. Interaction between androgens and AR initiates and drives progression of BPH by altering expression of factors involved in EMT [9, 23]. Interestingly, we found that GV1001 peptide bound to AR expressed by prostatic epithelial cells and stromal cells, and that blockade of AR with an anti-AR Ab reduced the binding activity of GV1001 to AR.

Since androgen-responsive genes (ARGs) play a role in prostate cell proliferation, communication, and differentiation [31], it is thought that the GV1001-AR interaction interferes with proliferation and DNA synthesis in prostate cells (Figure 1). In addition, reduced prostatic hyperplasia and hypertrophy (Figure 3A) due to the inhibitory effects of GV1001 were exerted via its interaction with AR in the prostate. In addition to AR, gonadotropin-releasing hormone receptors (GnRHR) in the prostate exert a local effect on tissue growth during BPH development and progression. For this reason, antagonists of GnRHR are approved and used widely for therapy of BPH and prostate cancer (PCa) [32]. Even though not presented here, we found that GV1001 also binds to GnRHR (data not shown). Taken together, the results suggest that GV1001 is a potential therapeutic drug for BPH because it not only regulates signals via AR but also via GnRHR.

According to Lee et al., GV1001 can interact with HSPs such as HSP70 and HSP90 [21]. It is already known that AR is a client protein of several HSPs [33]. AR is expressed by both epithelial and stromal tissues in the prostate, and ligand-unbound AR resides in the cytoplasm where it interacts with a complex of chaperones such as HSP40, HSP70, and HSP90, and co-chaperones [34]. Binding of HSP40-HSP70 to AR is followed by binding of HSP90 to AR, to generate a conformational structure with high affinity for DHT; the DHT/AR interaction leads to phosphorylation and dimerization of AR. The AR dimer is trafficked to the nucleus and facilitates transcriptional regulation of target genes [21]. Therefore, GV1001 appears to exert anti-BPH effects by competing with DHT for binding to AR, as well as inhibiting conformational changes in the AR after binding to HSPs. Therefore, it is thought that GV1001 effectively disrupts the interaction between AR and HSPs during BPH development and progression.

Normal prostate homeostasis is regulated by androgens and is characterized by slow growth kinetics, which are controlled by a balance between the rates of cell proliferation and apoptosis [35]. Loss of apoptosis control results in an increased proliferation/apoptosis ratio, leading to BPH [35, 36]. AR in the prostate promote development of BPH by regulating proliferation of prostate cells [9]. Expression of ARG is up-regulated from ~2-fold to ~6-fold in the prostate epithelium of BPH cases when compared with adjacent normal cells [37]. Also, AR expression and staining intensity in the nuclei of epithelial and stromal cells is significantly higher in BPH than in normal prostate [38]. AR expression in the prostate of TE-treated mice increased; however, this was reversed by GV1001 or finasteride (Supplementary Figure 6). Further studies should examine how GV1001 down-regulates expression of ARs.

EMT is a physiological process that allows polarized epithelial cells, which normally interact with the basement membrane, to undergo multiple biochemical changes and acquire a mesenchymal cell phenotype; this is characterized by enhanced migratory capacity, invasiveness, elevated resistance to apoptosis, and greatly increased production of extracellular matrix components [26]. Although EMT is associated mainly with cancer progression (i.e., migration, invasion, and metastasis) [39], it also contributes to BPH [23, 40]. EMT is closely related to TGF-β and AR signaling. ARs induce EMT by activating the Snail transcription factor or by repressing E-cadherin. Activation of the Snail, ZEB-1, or Smad transcription factors initiates expression of mesenchymal genes [24]. In particular, TGB-β is a key mediator of EMT; also, TGF-β controls the rate of apoptosis, which affects cell proliferation, differentiation, and apoptosis signaling [41]. Elevated expression of TGF-β deregulates apoptosis, thereby contributing to BPH; studies show that overexpression TGF-β and increased signaling among the epithelial and stromal components in BPH impact the growth dynamic of the benign gland [35, 36]. Thus, we asked whether prostatic stromal and epithelial cells affect each other with respect to cytokines production and proliferation. Taken together, we found that GV1001 downregulated expression of TGF-β in mitomycin C-treated RWPE-1 and WPE-NA22 when co-cultured with WPMY-1. However, neither production of IL-1β and EGF nor cell proliferation was affected (Supplementary Figure 7).

Fibrosis, which occurs in a number of epithelial tissues, is mediated by inflammatory cells and fibroblasts that release inflammatory signals and extracellular matrix (ECM) components such as collagens, elastin, and tenascin [26]. Furthermore, EMT plays a role in the genesis of fibroblasts during organ fibrosis [42]. In particular, TGF-β induces smooth muscle differentiation in addition to accumulation of ECM proteins [43]. Figure 3D shows reduced immune cell infiltration in the prostate of BPH-induced mice after GV1001 treatment. Indeed, the anti-inflammatory effects of GV1001 have been reported in IRI mice and in Aβ-treated neural stem cells [44, 45]. Moreover, GV1001 inhibited DHT-induced production of inflammation-related factors such as TNF-α in PBMCs (Figure 5). As a result of reduced inflammation, EMT and fibrosis were abrogated after GV1001 treatment. In our previous study, we observed that GV1001 reduced tumor size by decreasing fibrotic lesions in mice bearing pancreatic tumors [18]. The anti-fibrotic effect of GV1001 is now under investigation.

In conclusion, GV1001 relieves BPH by suppressing proliferation of prostatic epithelial and stromal cells via interaction with AR, thereby reducing the size of the prostate, reducing PSA levels, inhibiting immune cell infiltration, and preventing TGF-β-mediated EMT.

## MATERIALS AND METHODS

### Cell culture

Human prostatic epithelial cell lines RWPE-1 and WPE1-NA22, and prostatic stromal myofibroblast cell line WPMY-1, were purchased from the American Type Culture Collection (ATCC, Manassas, VA, USA). RWPE-1 and WPE1-NA22 cells were maintained in keratinocyte serum-free medium (K-SFM; GIBCO, Grand Island, NY, USA) supplemented with bovine pituitary extract (0.05 mg/ml, BPE), human recombinant epidermal growth factor (5 ng/ml, EGF), and 10% heat-inactivated fetal bovine serum (FBS; Hyclone, Logan, UT, USA). WPMY-1 cells were maintained in DMEM (ATCC) containing 5% FBS (Hyclone). Additionally, all media were supplemented with 100 units/ml of penicillin and 100 μg/ml of streptomycin (Welgene, Daegu, Korea). Cells were maintained in continuous log phase of growth at 37° C in a humidified atmosphere containing 5% CO_2_.

### GV1001

GV1001 was synthesized and kindly provided by Sang Jae Kim (president of GemVax and KAEL).

### Cell proliferation assay

RWPE-1 (1 × 10^4^), WPE1-NA22 (1 × 10^4^) and WPMY-1 (2.5 × 10^3^) cells were seeded in 96 well plates. After 16 h, cells were treated with DHT (25 nM; Sigma, St. Louis, MO, USA) and GV1001 (100 μM; GemVax and KAEL, Sungnam, Korea) for 48 h. After removal of the medium by suction, cells were incubated with Cell Counting Kit-8 assay (CCK-8; Dojindo Laboratories, Kumamoto, Japan) solution for 1–4 h. Optical density was measured at 450 nm in a spectrophotometer (Molecular Devices, Sunnyvale, CA, USA) according to the manufacturer’s instructions.

### Flow cytometry

For cell cycle analysis, RWPE-1, WPE1-NA22, and WPMY-1 cells were treated with DHT and GV1001 for 48 h and washed with PBS. Then, cells were re-suspended in 70% ethanol (drop by drop) and incubated at -20° C overnight. After washing with FACS buffer (PBS containing 0.5% BSA and 0.1% sodium azide), cells were suspended in propidium iodide (PI)/RNase staining buffer (BD Pharmingen, San Diego, CA, USA) for 15 min at room temperature. To examine the interaction between GV1001 and AR, fixed/permeabilized RWPE-1 and WPMY-1 cells were incubated for 1 h at room temperature with 1 μg of anti-AR (Santa Cruz Biotechnology, Dallas, TX, USA) antibody or an isotype control with rotation. Next, cells were stained for 30 min on ice with FITC-conjugated GV1001 and washed with FACS buffer. Flow cytometry was performed using a FACSCalibur^TM^ cytometer (BD Biosciences, San Jose, CA, USA) and data were analyzed using FlowJo software (BD Biosciences, Franklin Lakes, NJ, USA).

### Analysis of cellular uptake by fluorescence microscopy

Cells were seeded overnight on 12 mm cover slips. After washing with PBS, GV-FITC (100 μM) or FITC (Sigma) was added for 1 h at 37° C in a humidified atmosphere containing 5% CO_2_. Cells were fixed for 15 min at 4° C with 2% paraformaldehyde (PFA). After staining with mounting-DAPI medium (ImmunoBioscience, Mukilteo, WA, USA), cells were analyzed by fluorescence microscopy (EVOS M5000; Invitrogen, Carlsbad, CA, USA).

### Immunoprecipitation

RWPE-1 and WPMY-1 cells were treated with GV-FITC (100 μM) for 1 h at 37° C in a humidified atmosphere containing 5% CO_2_. For co-immunoprecipitation, an AR antibody was conjugated to Dynabeads Protein G (Invitrogen). RWPE-1 and WPMY-1 cell lysates were prepared and incubated with antibody-conjugated Dynabeads. To detect the GV-FITC bound to AR, eluted samples were detected using Molecular Devices Fluorometer at an excitation wavelength of 488 nm and an emission wavelength of 525 nm.

### Animals

C57BL/6J male mice (20–25 g; 7–8 weeks old; n=7) were mated and housed under specific pathogen free conditions at the animal facility in the Seoul National University College of Medicine. All experiments were reviewed and approved by the Institutional Animal Care and Use Committee of Seoul National University (Animal approve #SNU-100428-3-4). In the mouse BPH model, the micro-osmotic pump (ALZET, Cupertino, CA, USA) was filled with TEs (EVER Pharma, GmbH, Germany) diluted in corn oil (Sigma) and implanted into the caudal thigh muscle. The pump released TE (2.5 mg/mouse, 0.11 μl/h) over a period of 2 weeks. Next, mice received an intraperitoneal injection of GV1001 (250 μg/head) or finasteride (100 mg/kg) for 2 weeks. Finally, mice were anesthetized with Zoletil (25 mg/kg) and xylazine (10 mg/kg) and blood and prostate were collected.

### Immunohistochemistry

Prostate tissue was isolated from mice and immediately embedded in paraffin after fixation with 4% PFA at 4° C. Paraffin-embedded tissues were sectioned (5 μm thick), and de-paraffinized, and hydrated. After staining with hematoxylin and eosin (H&E) or Masson’s Trichrome (Sigma), tissues were dehydrated and mounted. The bar graph of fibrosis area was computed by dividing the area of the positive region (stained blue) by that of the fibromuscular stroma. Antigenic retrieval was performed by tissues heating hydrated tissue in 0.1 M citrate buffer (pH 6.0) in a microwave. After blocking endogenous peroxidase with H_2_O_2_ and inhibiting nonspecific signals with 5% goat serum, sections were incubated at 4° C overnight in a humidified chamber with a primary antibody specific for Ki67 (Abcam, Cambridge, UK). Then, sections were incubated for 1 h at room temperature with a biotinylated anti-rabbit antibody (Vector Laboratories, Burlingame, CA, USA). Next, sections were covered with ABC solution (Vector Laboratories) for 30 min. A DAB kit (Vector Laboratories) was used for chromogenic detection. Subsequent to dehydration and clearing, sections were mounted in DPX (Fluka, St. Louis, MO, USA) and observed under a light microscope (Olympus, Center Valley, PA, USA).

### Western blot analysis

Prostate tissue was homogenized in lysis buffer, and total protein was quantified in a bicinchoninic acid assay. Equal amounts of protein were resolved in 10% polyacrylamide gels and transferred to nitrocellulose membranes. After blocking with 5% nonfat milk, membranes were incubated overnight at 4° C with anti-PCNA (Santa Cruz Biotechnology), anti-PSA (Abcam), and β-actin (Santa Cruz Biotechnology) antibodies. After incubating with horseradish peroxidase-conjugated anti-rabbit or anti-mouse IgG (Cell Signaling), immunoreactive proteins were visualized using the ECL detection system (Amersham Biosciences Corp., Piscataway, NJ, USA).

### Immunoassay

Blood was collected from the intra-orbital plexus into a heparinized capillary tube and centrifuged at 12000 × g for 30 min at 4° C. The plasma in the upper layer was stored at -80° C until use. The concentration of plasma testosterone and DHT was measured using an EIA kit (CUSABIO, Wuhan, China). Prostate glands were excised, frozen rapidly in liquid nitrogen, and homogenized in lysis buffer. The concentration of TGF-β in prostatic lysates was measured using an ELISA kit (eBioscience, San Diego, CA, USA). The final concentration of TGF-β in the prostates was normalized against the amounts of total protein in prostatic tissue lysates. EIA and ELISA were performed according to the manufacturers’ instructions.

### Immunohistochemistry-Fr

Fixed tissues were embedded in paraffin and sectioned (4 μm thick). After deparaffinization and hydration, antigen retrieval was performed to heat the antigens in a microwave oven in 0.1 M citrate buffer (pH 6.0). Tissue sections were incubated at 4° C overnight in a humidified chamber with primary antibodies specific for Snail + Slug (1:100; Abcam), Vimentin (1:50; Santa Cruz), N-Cadherin (1:100; Santa Cruz), and E-cadherin (1:100; Santa Cruz), followed by incubation for 1 h at room temperature with Alexa Fluor 488 goat anti-rabbit IgG (H+L) (1:150; Invitrogen) for Snail + Slug, and E-cadherin and Alexa Fluor 488 donkey anti-mouse IgG (H+L) (1:150; Invitrogen) for Vimentin and N-cadherin. After staining, tissue sections were mounted with DAPI (ImmunoBioscience, Mukilteo, WA, USA) and visualized using EVOS M5000 (Invitrogen).

### Human cytokine array

The research performed in this study followed the tenets of the Declaration of Helsinki and was approved by the Institutional Ethics Committee of Seoul National University Hospital. After written informed consent was obtained, PBMCs were obtained from 20 healthy individuals by density gradient centrifugation on Ficoll-Paque^TM^ PLUS (Amersham Pharmacia Biotech, Piscataway, NJ, USA). The pellet was harvested and re-suspended for 5 min at 37° C in red blood cell lysis buffer (Sigma). To investigate cytokine production, PBMCs (2.5 × 10^6^ cells/ml) were treated with (or not) GV1001 (100 μM) and DHT (25 nM) for 24 h. Culture supernatant was assessed using Human Cytokine Array Q1 (RayBiotec, Norcross, GA, USA). Data were scanned by a gene microarray laser scanner and densitometry and analysis were performed using Q-Analyzer.

### Statistical analysis

Data are expressed as the mean ± SD of three independent experiments. To compare three or more groups, data were analyzed using a t-test or one-way analysis of variance, followed by Newman-Keuls multiple comparison tests. P-value < 0.05 was considered statistically significant. Statistical tests were carried out using GraphPad InStat (GraphPad Software, San Diego, CA, USA).

## Supplementary Material

Supplementary Figures
