# Supplementary material for: GV1001 interacts with androgen receptor to inhibit prostate cell proliferation in benign prostatic hyperplasia by regulating expression of molecules related to epithelial-mesenchymal transition
Source: Aging (Albany NY). 2021 Feb 4;13(3):3202–17. doi: 10.18632/aging.202242 (PMC7906190; doi:10.18632/aging.202242)
Supplement: Supplementary Figures [file aging-13-202242-s001.pdf]

SUPPLEMENTARY FIGURES

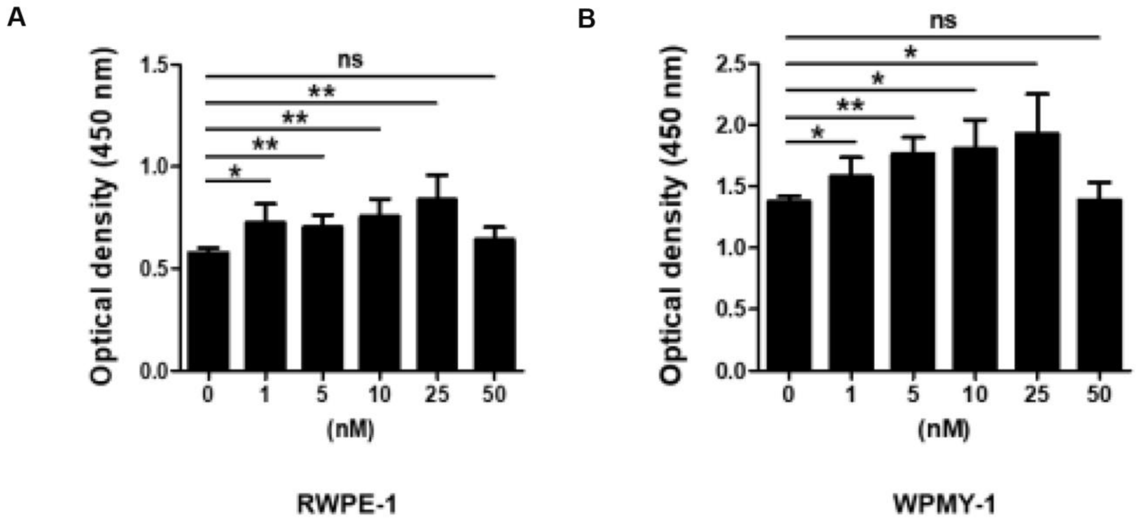

Supplementary Figure 1. Proliferation of prostatic epithelial and stromal cells treated with DHT. (**A**) Prostatic epithelial cell line RWPE-1 and (**B**) prostatic stromal cell line WPMY-1 were exposed to various concentrations of DHT for 48 h, and cell proliferation was examined in a CCK-8 assay. Proliferation increased in a dose-dependent manner. *p<0.05, **p<0.01.

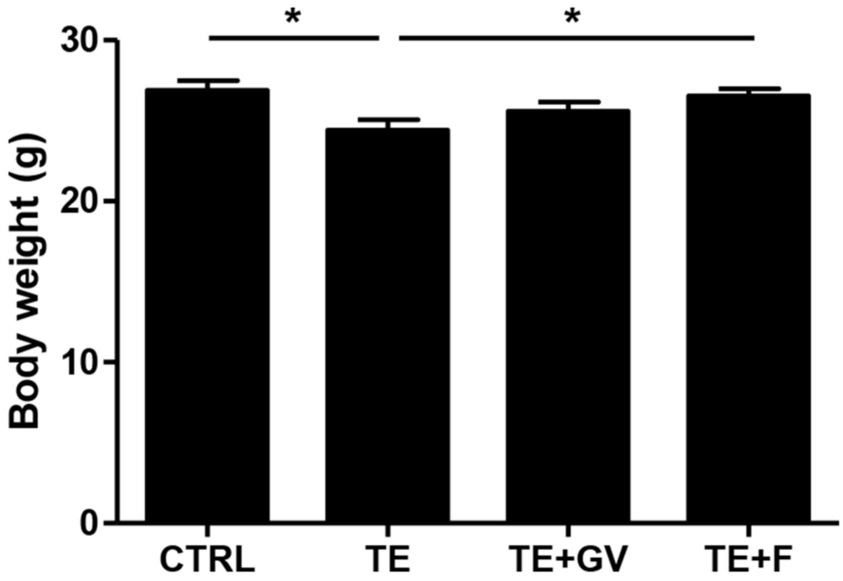

Supplementary Figure 2. Body weight of TE-induced BPH mice. BPH mice received GV1001 (GV, 250 μg/head) or Finasteride (F, 100 mg/kg) for 2 weeks. Body weight was measured at the end of the experiment. *p<0.05.

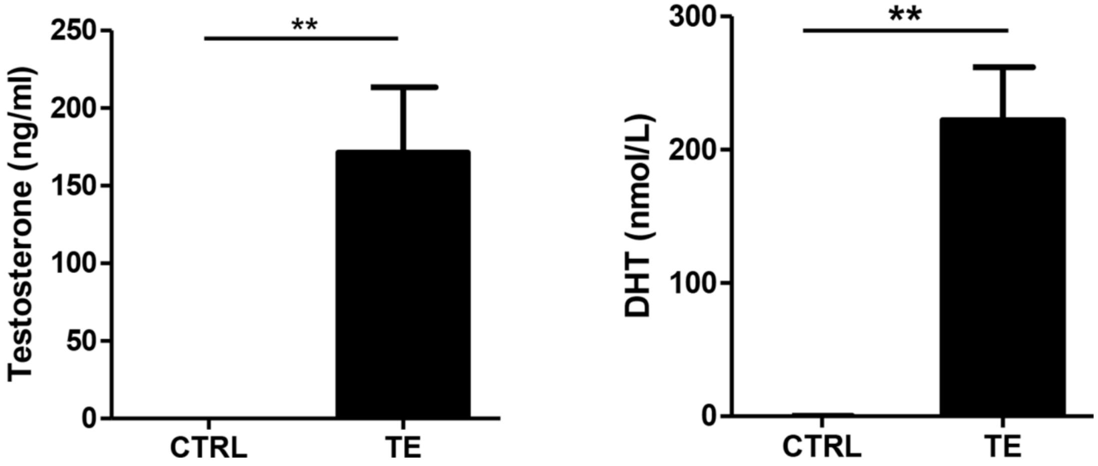

Supplementary Figure 3. Testosterone and DHT levels in TE-induced BPH mice. Mice were injected with TE via an osmotic pump (TE release; 0.11 µl/h) over a period of 2 weeks. **p<0.01.

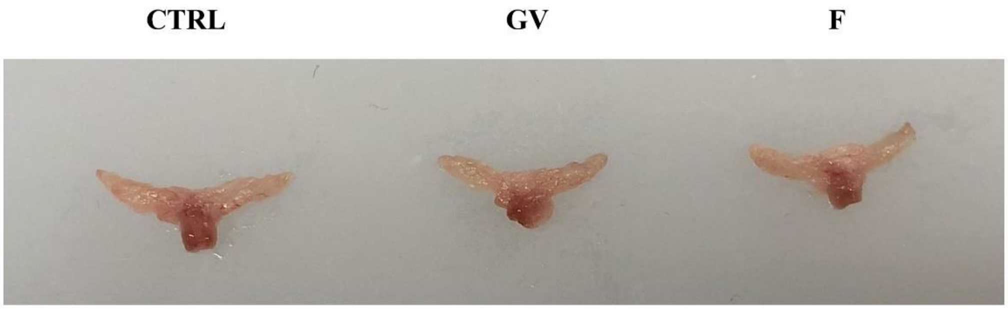

Supplementary Figure 4. GV1001 and Finasteride have no effect in BPH mice. BPH mice received GV1001 (GV, 250 µg/head) or Finasteride (F, 100 mg/kg) for 2 weeks. Prostates were then excised for examination.

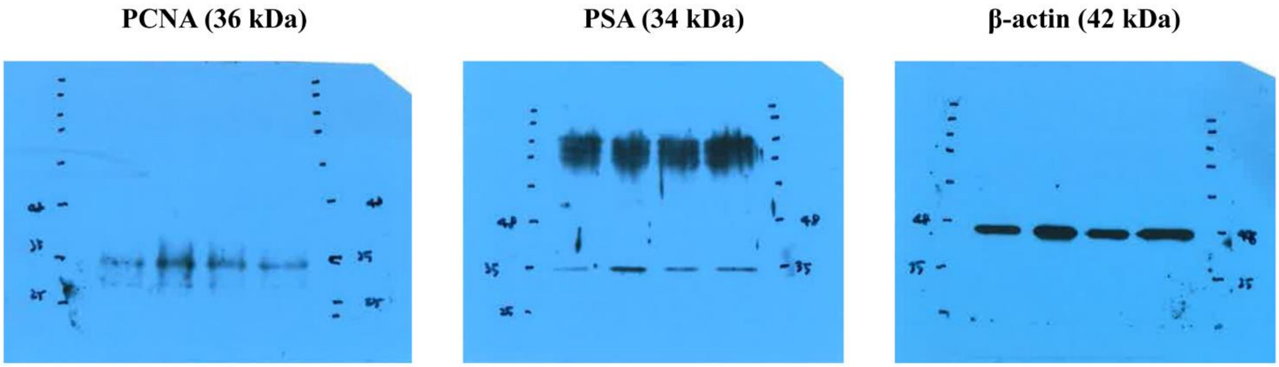

Supplementary Figure 5. Full blot from Figure 3F.

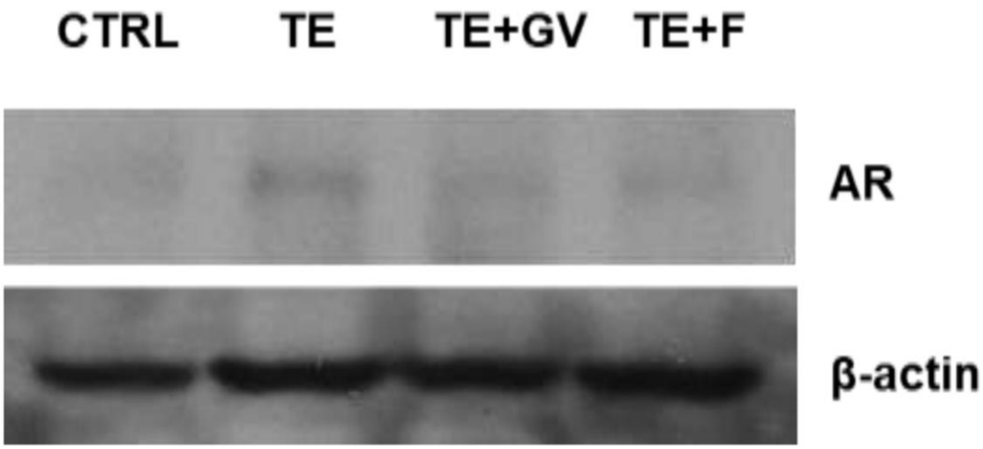

Supplementary Figure 6. GV1001-mediated downregulation of androgen receptor expression in the prostate of BPH mice. Expression of AR in testosterone-induced BPH mice was measured by immunoblotting after treatment with GV1001 or Finasteride**.**

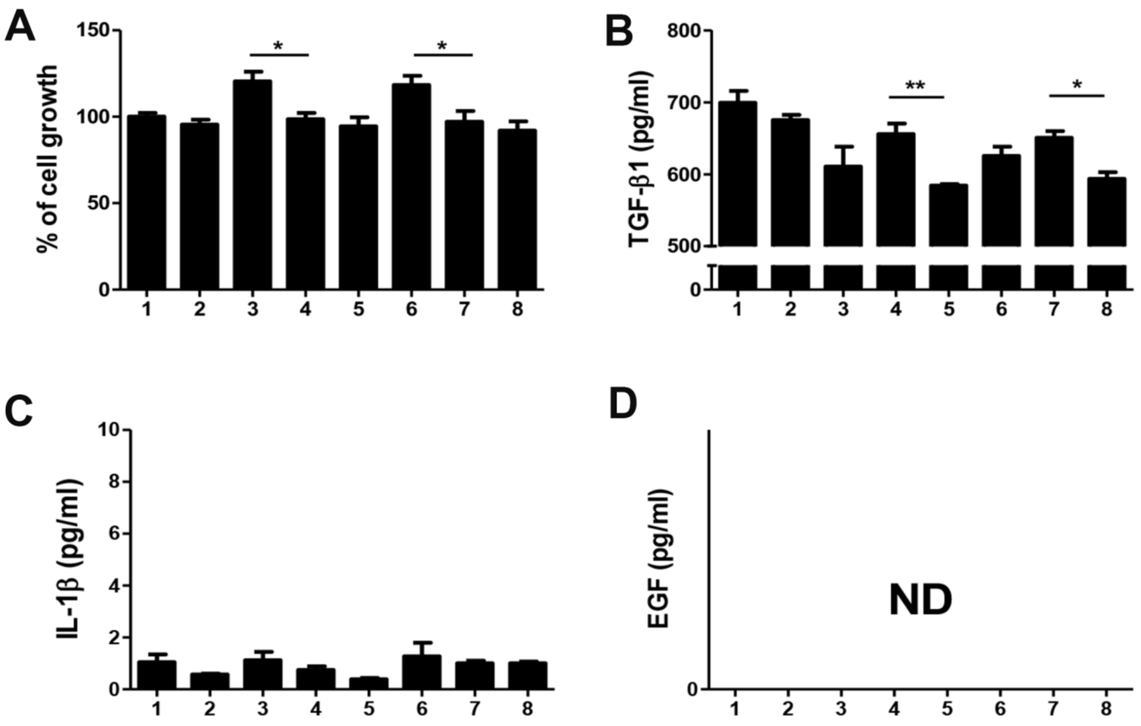

Supplementary Figure 7. GV1001-mediated reduction of TGF-β1 production in mitomycin C-treated RWPE-1 and WPE1-NA22 cells co-cultured with WPMY-1. RWPE-1 and WPE1-NA22 cells were treated with mitomycin C (10 μg/ml) for 2 h. Then, cells were co-cultured with WPMY-1 for 48 h. Groups are as follows: 1, WPMY-1 only (5×10^5 cells); 2, WPMY-1 + GV1001 (100 μM); 3, WPMY-1 + RWPE-1 (2.5×10^5 cells); 4, WPMY-1 + Mitomycin C-treated RWPE-1; 5, WPMY-1 + Mitomycin C-treated RWPE-1 + GV1001 (100 μM); 6, WPMY-1 + WPE1-NA22 (2.5×10^5 cells); 7, WPMY-1 + Mitomycin C-treated WPE1-NA22; and 8, WPMY-1 + Mitomycin C-treated WPE1-NA22 + GV1001 (100 μM). (**A**) Percent cell growth was calculated as follows: % cell growth = (the number of WPMY-1 cells/the following number as above) × 100. (**B**–**D**) Supernatants were collected, and the quantity of IL-1β, EGF, and TGF-β1 was measured by ELISA. Each sample was measured in triplicate, and results are representative of three independent experiments. Data are presented as the mean ± SD. *$p<0.05$, **$p<0.01$.
